# Weight stigma in patients with overweight and obesity: validation of the Italian Weight Self-Stigma Questionnaire (WSSQ)

**DOI:** 10.1007/s40519-022-01385-8

**Published:** 2022-03-15

**Authors:** Alessandro Alberto Rossi, Gian Mauro Manzoni, Giada Pietrabissa, Daniele Di Pauli, Stefania Mannarini, Gianluca Castelnuovo

**Affiliations:** 1 Department of Philosophy, Sociology, Education, and Applied Psychology, Section of Applied Psychology, University of Padova, Padua, Italy; 2 Interdepartmental Center for Family Research, Department of Philosophy, Sociology, Education, and Applied Psychology, Section of Applied Psychology, University of Padova, Padua, Italy; 3 Psychology Research Laboratory, Ospedale San Giuseppe, IRCCS, Istituto Auxologico Italiano, Verbania, Italy; 4 Faculty of Psychology, eCampus University, Novedrate, Como Italy; 5 Department of Psychology, Catholic University of Milan, Milan, Italy; 6 Independent Researcher, Verona, Italy

**Keywords:** Obesity, Stigma, Weight-related self-stigma, Internalized weight stigma, Body image

## Abstract

**Purpose:**

This study aimed to explore the factorial structure of the Italian Weight Self-Stigma Questionnaire (WSSQ) (Study1); and to test structural validity, internal consistency, test–retest reliability, and measurement invariance of the questionnaire across gender, Body Mass Index (BMI), age and occurrence of previous hospitalization for obesity (Study2).

**Methods:**

At admission into a hospital-based program for weight reduction and rehabilitation, 150 inpatients with overweight/obesity (68% females) completed the WSSQ (Study1). In Study2, in addition to the WSSQ, 446 inpatients (61.9% females) completed the Weight Bias Internalization Scale (WBIS), the Body Uneasiness Test (BUT), and the Center for Epidemiologic Studies Depression Scale (CES-D). A subsample of 40 patients also re-completed the WSSQ at discharge from the hospital.

**Results:**

The Italian WSSQ showed good overlap with the original factorial structure (Study1) and results were confirmed in Study2. Test–retest reliability and convergent validity showed adequate values. Measurement invariance revealed that WSSQ was perfectly invariant across both BMI and the occurrence of previous hospitalizations for obesity. In both studies, the internal consistency of the questionnaire was deemed acceptable.

**Conclusions:**

The Italian WSSQ is a valid, reliable, and invariant tool for the assessment of weight-related self-stigma among patients with overweight/obesity. Future studies should assess its longitudinal invariance as well as its responsiveness to weight reduction treatments.

**Level of evidence:**

V, descriptive study.

## Introduction

Overweight and obesity are chronic conditions [1] that affect over 600 million people worldwide [2–5]—including the elderly population [6–9].

Besides these alarming rates, several studies showed that the prevalence of weight stigmatization has rapidly increased during the last 20 years [10–14]. Indeed, people with overweight and obesity are regularly exposed to weight-related negative bias and discrimination in everyday life [12, 15] such as workplace, educational, and health care settings, in the media as well as in interpersonal relationships [16]. As a consequence of this continuous exposure to weight stigma, people with overweight and obesity tend to internalize these biases [17–19].

The internalization of weight sigma (IWS) leads individuals with overweight and/or obesity to self-stigmatize themselves, thus confirming main negative weight stereotypes [13, 18]—such as failure to keep self-control [20], blame, incompetence [21], and lack of self-discipline—further increasing their risk of being socially devaluated and/or rejected [12, 15, 16]. Moreover, IWS plays a fundamental role in non-adherence to weight loss programs [22]—which in turn contributes to increasing stigma and further promotes weight gain—as is was also observed in patients undergoing bariatric surgery [23]. IWS is also associated with adverse health outcomes including cardiovascular diseases, hypertension, osteoarthritis, and diabetes [24, 25], and it is also strongly related to poor quality of life as well as psychological/psychiatric disorders, such as depression, anxiety, distress, somatization, and eating problems [17, 18, 26–29].

Despite multidisciplinary interventions have been developed to improve weight loss and to reduce IWS [30–34], the extent to which weight self-stigma affects health outcomes is still unclear [26, 27]. To fill the knowledge gaps in the particular field, the Weight Self-Stigma Questionnaire (WSSQ), was shown to be a reliable and valid tool to assess IWS and to detect the impact of effective interventions targeting weight-related stigma [17].

The WSSQ was first developed and validated in two American samples (*n* = 169) of participants with overweight/obesity (non-treatment-seeking and treatment-seeking). The resulting two main factors (“self-devaluation” and “fear of enacted stigma”) described by the WSSQ showed good reliability and validity indices as well as an adequate sensitivity to change in subjects seeking treatment for IWS reduction, as well as a good sensitivity to change and temporal stability. Good psychometric proprieties of the WSSQ—such as convergent validity, reliability, and predictive values for psychological variables—were confirmed in a later study [13] Moreover, the scale showed associations with clinical and/or subclinical conditions such as anxiety, depression, Quality of Life (QoL) and self-esteem [17].

The WSSQ was adapted in German [19], Arabic [35], and French [36] language. The German version [19] was validated in a sample of 94 adult outpatients with severe obesity demonstrating good psychometrical proprieties. Hain and colleagues also showed the impact of feelings of guilt (related to body image), BMI (≥ 50), and dissociative symptoms in the prediction of IWS [19]. The Arabic version [35] was validated on a sample of 170 Iranian women with overweight/obesity and showed good validity and internal consistency indices. Moreover, the authors evaluated the association between IWS, QoL, and psychological distress. Results confirmed the negative impact of IWS on both psychological distress and QoL, suggesting that IWS might represent a barrier for subjects with obesity to undertake health-promoting attitudes and behaviors. However, neither for the German study nor for the Arabic validation of the WSSQ any factor analysis (exploratory or confirmatory) was conducted to assess the factorial structure of the questionnaire. Instead, a confirmatory factor analysis (CFA) approach was employed to validate the French version of WSSQ [36]. The authors tested the factorial structure of the questionnaire in a sample of 156 adolescents with overweight/obesity, and findings revealed a good of model fit indices and reliability. Maïano and colleagues also assessed the correlation (convergent validity) between IWS, self‑esteem, anxiety, depression, fear of appearance evaluation, and tendency to eating‑related pathology. Results revealed an association between IWS and all the above psychological constructs. In addition, the authors tested the measurement invariance of the tool across gender using a MIMIC-based (Multiple Indicators Multiple Causes) approach, which revealed an influence of participant gender on WSSQ response. Notably, in line with the literature, none of the above validation studies showed associations between IWS and BMI, suggesting that IWS is complex phenomenon that goes beyond the mere individuals’ BMI [18]. Given that the IWS is a well-known risk-factor among those negatively influencing the individuals’ psychological health [37] in individuals with overweight and obesity, it is important to assess it both in the clinical and research fields.

Since there is no standardized validated version of the WSSQ available for the Italian population, the present study aimed to explore the factorial structure of the Italian version of the WSSQ (Study1), and to assess its structural validity, internal consistency, test–retest reliability, and measurement invariance across main socio-demographics characteristics (Study2).

## Study1

### Methods

#### Translation and cross-cultural adaptation

The WSSQ was independently translated by two Italian experts in the field and back-translated into English by an independent translator—to guarantee conceptual equivalency [38]. The questionnaire was also administered to 10 subjects with overweight/obesity—who did not enter the analysis—to assess whether the items were understandable by the target population. No adjustments were needed.

#### Participants and procedure

According to the most commons rule-of-thumb for Exploratory Factor Analysis (EFA) [39], the sample was composed by 150 adult inpatients [48 males (32%) and 102 females (68%)] with overweight/obesity—aged from 18 to 78 years (*mean* = 50.34, *SD* = 14.457). Participants were recruited from the IRCCS Istituto Auxologico Italiano, San Giuseppe Hospital, Verbania (Italy), during their first week of a 1-month hospital-based program for weight reduction and rehabilitation. Inclusion criteria were: (A) being a native Italian speaker; (B) being over 18 years; (C) signing written and informed consent. Exclusion criteria were: (D) illiteracy; (E) inability to complete the assessment due to vision and/or cognitive impairments. All participants completed a self-report demographic and anthropometric measures form and the Italian WSSQ. Sample descriptive statistics are displayed in Table 1.

**Table 1:** Study1 and Study2: sample descriptive statistics

	Study1			Study2		
	Overall (*n* = 150)	Male (*n* = 48)	Female (*n* = 102)	Overall (*n* = 446)	Male (*n* = 170)	Female (*n* = 276)
Weight in kg (mean; SD)	120.9 (24.6)	135.1 (25.6)	114.3 (21.2)	114.4 (24.8)	128.1 (24.4)	105.9 (20.9)
Height in m (mean; SD)	1.64 (0.09)	1.73 (0.06)	1.60 (0.07)	1.64 (0.11)	1.73 (0.08)	1.59 (0.07)
BMI (mean; SD)	44.67 (7.68)	44.79 (7.77)	44.61 (7.68)	42.29 (7.57)	42.55 (7.26)	42.14 (7.76)
Age (mean; SD)	50.3 (14.5)	52.8 (14.5)	49.2 (14.3)	51.7 (13.1)	49.6 (13.2)	52.9 (12.9)
Relationship status (*n*; %)						
Single/never married	46 (30.7%)	15 (31.3%)	31 (30.4%)	128 (28.7%)	62 (36.5%)	66 (23.9%)
Married/in a relationship	63 (42.0%)	22 (45.8%)	41 (40.2%)	236 (52.9%)	84 (49.4%)	152 (55.1%)
Separated/divorced	32 (21.3%)	10 (20.8%)	22 (21.6%)	54 (12.1%)	19 (11.2%)	35 (12.7%)
Widowed	9 (6.0%)	1 (2.1%)	8 (7.8%)	28 (6.3%)	5 (2.9%)	23 (8.3%)
Education status (*n*; %)						
No education	0 (0.0%)	0 (0.0%)	0 (0.0%)	5 (1.1%)	1 (0.6%)	4 (1.4%)
Primary school	10 (6.7%)	2 (4.2%)	8 (7.8%)	72 (16.1%)	20 (11.8%)	52 (18.8%)
Secondary school	74 (49.3%)	21 (43.8%)	53 (52.0%)	184 (41.3%)	88 (51.8%)	96 (34.8%)
High school	54 (36.0%)	19 (39.6%)	35 (34.3%)	146 (32.7%)	48 (28.2%)	98 (35.5%)
Bachelor degree	12 (8.0%)	6 (12.5%)	6 (5.9%)	38 (8.5%)	13 (7.6%)	25 (9.1%)
Postgraduate	0 (0.0%)	0 (0.0%)	0 (0.0%)	1 (0.2%)	0 (0.0%)	1 (0.4%)
Employment status (*n*; %)						
Full-time	92 (61.3%)	26 (54.2%)	66 (64.7%)	207 (46.4%)	81 (47.6%)	126 (45.7%)
Part-time	9(6.0%)	0 (0.0%)	9 (8.8%)	85 (19.1%)	34 (20.0%)	51 (18.5%)
Unemployed	20 (13.3%)	10 (20.8%)	10 (9.8%)	72 (16.1%)	23 (13.5%)	49 (17.8%)
Student	8 (5.3%)	1 (2.1%)	7 (6.9%)	9 (2.0%)	7 (4.1%)	2 (0.7%)
Retried	21 (14.0%)	11 (22.9%)	10 (9.8%)	73 (16.4%)	25 (14.7%)	48 (17.4%)
Past hospitalization (*n*; %)						
Yes	62 (41.3%)	26 (54.2%)	36 (35.3%)	200 (44.8%)	96 (56.5%)	104 (37.7%)
No	88 (58.7%)	22 (45.8%)	66 (64.7%)	246 (54.2%)	74 (43.5%)	172 (62.3%)

Due to the strong different sample size in each group, Cramér’s V was performed to test potential association between ordinal/categorical variables. Independent sample *t*-test was performed to assess potential differences between continuous almost-normal distributables. *n* = no difference between males and female

### Measures

#### Demographic and anthropometric measures

Participants were asked for demographic information (Table 1) including gender, civil status, educational level, work status, and the occurrence of previous hospitalization(s) for obesity and weight reduction, as well as to provide anthropometric measures, such as age, height, and weight (used to calculate their BMI).

#### Weight Self‑Stigma Questionnaire

Participants completed the Italian version of the Weight Self-Stigma Questionnaire (WSSQ) [17]. It consists of 12 items rated on a five-point Likert scale (from 1 = strongly disagree to 5 = strongly agree) and provides scores in two domains (self-devaluation and fear of enacted domains). The first six items measure self-devaluation (Self-D), which is the continuous tendency to self-depreciating by attributing negative stigmatizing characteristics to his/her own [17]. An example of a representative item is: “*I became overweight because I’m a weak person*” (item#4) and “*I don’t have enough self-control to maintain a healthy weight*” (item#6). The following six items measure the fear of enacted stigma (FES), which refers to self-stigmatized thoughts [17, 40]. Representative items are: “*People discriminate against me because I’ve had weight problems*” (item#8) and “*Others will think I lack self-control because of my weight problems*” (item#10). Coefficient alphas of the original WSSQ were: Self-D = 0.812; FES = 0.869; total score = 0.878.

### Statistical analysis

Statistical analyses were performed with the R statistical software [41] with the following packages: psych [42] and MplusAutomation [43].

Item analysis was performed to check normality. Due to acceptable deviation from normality, Maximum Likelihood estimator (ML) has been used to perform the EFA [39, 44–46], with a Direct Oblimin rotation due to an expected correlation between factors. Factors were retained according to the 95th percentile of Parallel Analysis (PA) with 10,000 random correlation matrices—used to compute random data eigenvalues. The size of these random data eigenvalues was compared with the ones from the original observations. The best factor solution was determined by the number of eigenvalues that exceeded the corresponding values from the random dataset [47–49]. The factorability of the correlation matrix was evaluated with Bartlett’s Test of Sphericity and with Kaiser–Meyer–Olkin (KMO) measure of sampling adequacy. For the EFA to be considered appropriate, the above tests had to show significant results (*p* < 0.05) and values higher than 0.7–—respectively [39]. Lastly, Cronbach’s alpha was computed as measure of internal consistency with values higher than 0.7 deemed acceptable [39].

## Results

### Exploratory factor analysis of WSSQ

Item analysis reveals a univariate almost-normal distribution of all the indicators (Table 2). Absolute skewness ranged from |0.007| for item#11 to |1.033| for item#2: mean_sk_ = 0.03; SD_sk_ = 0.52. Absolute kurtosis ranged from |0.025| for item#2 to |1.313| for item#11: mean_k_ = − 0.96; SD_k_ = 0.38.

**Table 2:** Study1: item translation (English/*Italian*) of the WSSQ, item descriptive statistics and the exploratory factor analysis (EFA)

		Descriptive statistics					EFA	
		Mean	Medn.	SD	Sk	*K*	Factor 1 (*λ*)	Factor 2 (*λ*)
1	I will always go back to being overweight	2.44	2	1.176	0.349	− 0.736	0.421*	0.034
	*Ritornerò sempre a essere in sovrappeso*							
2	I caused my weight problems	3.85	4	1.319	− 0.951	− 0.254	0.541*	− 0.203*
	*Ho causato io i miei problemi di peso*							
3	I feel guilty because of my weight problems	3.41	4	1.289	− 0.333	− 0.950	0.753*	− 0.088
	*Mi sento in colpa a causa dei miei problemi di peso*							
4	I became overweight because I am a weak person	2.76	3	1.383	0.178	− 1.210	0.801*	0.133
	*Sono diventato in sovrappeso perché sono una persona debole*							
5	I would never have any problems with weight if I were stronger	3.10	3	1.408	− 0.124	− 1.223	0.788*	0.004
	*Se fossi più forte, non avrei mai avuto alcun problema con il peso*							
6	I do not have enough self-control to maintain a healthy weight	3.41	4	1.335	− .0474	− 0.988	0.577*	0.093
	*Non ho sufficiente auto-controllo per mantenere un peso salutare*							
7	I feel insecure about others’ opinions of me	2.81	3	1.330	0.148	− 1.101	0.221*	0.521*
	*Mi sento insicuro rispetto all’opinione altrui su di me*							
8	People discriminate against me because I have had weight problems	2.39	2	1.311	0.389	− 1.076	− 0.063	0.663*
	*La gente mi discrimina perché ho avuto problemi di peso*							
9	It is difficult for people who have not had weight problems to relate to me	2.19	2	1.237	0.611	− 0.792	− 0.188*	0.781*
	*Per le persone che non hanno avuto problemi di peso, è difficile relazionarsi con me*							
10	Others will think I lack self-control because of my weight problems	2.82	3	1.349	0.015	− 1.141	0.229	0.605*
	*Gli altri penseranno che mi manchi l’auto-controllo a causa dei miei problemi di peso*							
11	People think that I am to blame for my weight problems	2.81	3	1.374	0.003	− 1.259	0.225	0.624*
	*La gente pensa che io sia da incolpare per i miei problemi di peso*							
12	Others are ashamed to be around me because of my weight	2.05	1	1.318	0.857	− 0.625	0.040	0.627*
	*Gli altri si vergognano di starmi vicino a causa del mio peso*							

*M* = mean; *Medn.* = median *SD* = Standard deviation, *Sk* = skewness; *K* = kurtosis; *λ* = Factor loading

The highest factor loadings are highlighted in bold

*p < 0.05

Sample correlation matrix reveals two eigenvalues higher than 1 (i.e., I = 4.659; II = 1.945; III = 0.956) that were compared with PA’s random data eigenvalues (i.e., I = 1.622; II = 1.443; III = 1.326) allowing the extraction of two factors that explained the 55.04% of the overall variance. The first factor accounted for 38.83% of the variance, while the second one explained 16.21% of the variance. In line with the Kaiser–Meyer–Olkin’s test [KMO = 0.797], the Bartlett’s Test of Sphericity reached statistical significance: [*χ*^2^ (66) = 733.4, *p* < 0.001]—thus supporting the factorability of the correlation matrix.

As depicted in Table 2, the first six items—reflecting the original Self-D factor—loaded significantly on the first factor (*p* < 0.001): mean_loadings_ = 0.635; *SD*_loadings_ = 0.161, ranging from 0.389 (item#1) to 0.784 (item#3). However, item#2 and item#4 showed significant cross-loadings (respectively: *λ* = − 0.241; *λ* = − 0.156), albeit lower than 0.3. The last six items—reproducing the original FES—loaded significantly on the second factor (*p* < 0.001): mean_loadings_ = 0.631; *SD*_loadings_ = 0.07; ranging from 0.556 (item#10) to 0.746 (item#9). Still, four items (item#7, item#9, item#10 and item#11) showed significant cross-loadings (respectively: *λ* = 0.276; *λ* = − 0.170; *λ* = 0.233; *λ* = 0.213), although lower than 0.3. Furthermore, EFA revealed a significant correlation between the two factors: *r* = 0.444; *p* < 0.001. Cronbach’s alpha exhibited an acceptable internal consistency: Self-D: *α* = 0.815; FES: *α* = 0.827; total score: *α* = 0.852.

## Study2

### Methods

#### Participants and procedure

According to (A) Monte Carlo study simulation [50] and (B) the ratio of subjects-to-variables rule-of-thumb [45, 46], the sample was composed by 446 adult inpatients with overweight and obesity (BMI ≥ 35) [170 males (38.1%) and 276 females (61.9%)] aged from 18 to 81 years (*mean* = 51.69, *SD* = 13.09). Patients were enrolled at the IRCCS Istituto Auxologico Italiano, San Giuseppe Hospital, Verbania (Italy) and tested within 3 days from their admission into the 1-month hospital-based program for weight reduction and rehabilitation. Inclusion/exclusion criteria described in Study1 were applied. Complete descriptive statistics are presented in Table 1. Participants completed: (A) the demographic and anthropometric measures form—described in Study1; (B) the WSSQ; (C) the Weight Bias Internalization Scale (WBIS); (D) the Body Uneasiness Test (BUT); and (E) the Center for Epidemiologic Studies Depression Scale (CES-D). To assess test–retest reliability, a random subsample of 40 subjects was also retested at discharge from the hospital (after ± 28 days)—to ensure no memory effect [51]. During the hospitalization period, none of the patients received psychological interventions focused on the reduction of IWS.

### Measures

#### Weight Bias Internalization Scale (WBIS)

The WBIS [18, 52] is an 11-item—single-factor—self-report scale. Developed for people with overweight/obesity, the WBIS measures the agreement with self-directed negative statements about negative weight-related stereotypes. Higher scores are indicative of a higher weight-related bias own perception. In this study, the WBIS Cronbach’s alpha was 0.840.

#### Body Uneasiness Test (BUT)

The BUT [53] is a 34-item self-report questionnaire evaluating body uneasiness and concerns for physical appearance. Not designed for a specific target population, the BUT comprises a Global Severity Index (GSI) and 5 subscales: (I) fear regarding the body’s weight (Weight Phobia: WP), (II) over-concern related to physical appearance (Body Image Concerns: BICons), (III) body image-related avoidance behavior (Avoidance: A), (IV) compulsive checking of physical appearance (Compulsive Self-Monitoring: CSM), (V) estrangement feelings toward his/her own body (Depersonalization: Dep). In the present study, the BUT Cronbach’s alphas were: GSI = 0.960; WP = 0.849; BICons = 0.911; A = 0.873; CSM = 0.723; Dep = 0.867.

#### Center for Epidemiologic Studies Depression Scale (CES-D)

The CES-D [54, 55] is a self-report scale that assesses psychological and behavioral symptoms of depression. Developed for the general population, the CES-D is composed of 20-item loading on a single factor. Higher scores reflect greater depressive symptomatology. In the current study, the CES-D Cronbach’s alpha was 0.903.

### Statistical analysis

All statistical analyses were carried out with R statistical software [41] and the following packages: psych [42], MplusAutomation [43], lme4 [56], and irr [57].

Item analysis was performed to check normality. Due to a non-perfect normal distribution, Robust Maximum Likelihood estimator (MLR) was used to confirm the factorial structure of the Italian WSSQ. The MLR is a ‘robust’ variant of Maximum likelihood—which has many advantages over other estimators; i.e., computational efficiency [58]—providing robust standard errors and mean-adjusted *χ*^2^ statistics that are robust to non-normality and the violation of independence-of-observation assumption [45, 46, 58–61]. Model fit was tested using the *χ*^2^ statistic and the goodness-of-fit indices [45, 46, 62]: (A) the Root-Mean Square Error of Approximation (RMSEA) [63], (B) the Comparative Fit Index (CFI) [64] and (C) the Standard Root Mean square Residual (SRMR) [65]. A non-significant *χ*^2^ was desirable as indicating a better model fit [45, 46, 62]. The RMSEA expresses fit per degrees of freedom of the model, and values lower than 0.08 suggest an acceptable model fit [66, 67]. The CFI designates the amount of variance and covariance accounted by the model compared with a baseline, with values higher than 0.90 considered adequate [66, 67]. The SRMR represents the average discrepancy between the correlations observed in the input matrix and those predicted by the model [45, 65], and values lower than 0.08 are considered good [46, 66].

Multi-group CFAs were performed to test MI across (1) gender (male *vs.* female), (2) BMI (Class II obesity class *vs.* Class III obesity), (3) age (younger vs*.* older: grouped by the median split technique), and (4) previous hospitalization for obesity (occurrence vs*.* non-occurrence). MI entailed a series of hierarchical (nested) CFAs in which models parameters were consecutively constraint to be equals (invariants) across groups in a logically ordered and increasingly restrictive mode [45, 46, 67–70]. Nested model comparisons were conducted using (I) the Log-likelihood rescaled difference test (− 2ΔLL), (II) the test differences in absolute goodness-of-fit indices: CFI (ΔCFI > 0.01), RMSEA (ΔRMSEA > 0.015), and SRMR (ΔSRMR > 0.03) [71, 72]. The first step was to test the goodness-of-fit in the two groups, separately. The second step was to test the equivalence of the models (configural invariance). The third step (metric invariance) constrained the factor loadings to be equal across groups. The fourth step (scalar invariance) forced the equivalence of factor loadings and item’s intercepts across groups [45, 46, 67–70].

Moreover, items must provide information regarding the respondents: in other words, the item should be able to discriminate subjects with or without IWS. Therefore, following recommended guidelines [73], a series of independent sample *t*-tests were computed, and the Cohen’s *d* coefficient [74] was calculated to determine item discriminating power (IDP). These statistics refer to the magnitude to which the item discriminates the respondent’s level of the measured trait (low *vs.* high). A highly discriminating item divides clearly the respondents (*d* ≥ 0.80), a moderate discriminant item reveals regions of ambiguity (0.21 ≤ *d* ≤ 0.79) and a poorly discriminating item shows very small magnitude (*d* ≤ 0.20).

Moreover, since none of the participants received a psychological intervention to reduce IWS, temporal stability was assessed using a mixed-model repeated measure regression analysis accounting for random variability within individuals: a *non*-significant *p* value was expected.

Test–retest reliability was estimated using the two-way mixed intraclass correlation coefficient (ICC_consistency_) [39, 51, 75–77]

Pearson’s correlation was also computed to assess the convergent validity between internalized self-stigma (WSSQ), weight bias (WBIS), depressive symptomatology (CES-D), body uneasiness (BUT), and the individuals’ BMI.

## Results

### Model’s fit and psychometric proprieties: structural validity

Item analysis revealed a non-perfect univariate normal distribution of some indicators (Table 3). Absolute skewness ranged from |0.004| for item5 to |1.410| for item12: mean_sk_ = 0.29; SD_sk_ = 0.61. Absolute kurtosis ranged from |0.017| for item9 to |1.343| for item11: mean_k_ = -0.74; SD_k_ = 0.74.

**Table 3:** Study2: item descriptive statistics, confirmatory factor analysis (CFA), item discriminant power (IDP) and item temporal stability (ITS)

	Descriptive statistics							CFA			IDP		ITS	
	M	Medn.	SD	Sk	*K*	% Min	% Max	F1 (λ)	F2 (λ)	*R*^2^	*t*_*i*_	*d*	*t*_*p*_	*d*
Item1	2.24	2	1.105	0.462	− 0.603	33.6%	03.1%	0.338*	−	0.114*	− 8.86*	1.02	0.02^‡^	0.00
Item2	3.65	4	1.310	− 0.730	− 0.583	10.5%	33.0%	0.410*	−	0.168*	− 8.57*	1.00	− 0.53^‡^	0.11
Item3	3.30	3	1.368	− 0.366	− 1.048	15.9%	23.5%	0.597*	−	0.357*	− 16.60*	1.93	− 1.75^‡^	0.29
Item4	2.78	3	1.382	0.098	− 1.232	26.5%	13.5%	0.856*	−	0.733*	− 22.13*	2.56	0.16^‡^	0.02
Item5	2.94	3	1.451	0.004	− 1.335	24.7%	19.5%	0.846*	−	0.716*	− 19.23*	2.23	− 0.15^‡^	0.02
Item6	3.11	3	1.315	− 0.156	− 1.068	15.7%	17.5%	0.652*	−	0.425*	− 16.05*	1.86	− 1.40^‡^	0.18
Item7	2.64	3	1.405	0.232	− 1.271	31.4%	12.1%	−	0.668*	0.446*	− 25.13*	2.90	1.05^‡^	0.16
Item8	2.00	1	1.233	0.979	− 0.209	50.9%	05.2%	−	0.741*	0.549*	− 13.56*	1.56	0.46^‡^	0.06
Item9	1.96	1	1.229	1.052	− 0.017	53.4%	05.6%	−	0.621*	0.386*	− 11.23*	1.29	0.73^‡^	0.12
Item10	2.59	3	1.425	0.249	− 1.309	35.2%	11.7%	−	0.656*	0.430*	− 18.54*	2.14	0.90^‡^	0.17
Item11	2.66	3	1.423	0.188	− 1.343	32.5%	11.9%	−	0.644*	0.415*	− 17.45*	2.02	− 0.68^‡^	0.10
Item12	1.75	1	1.100	1.410	1.126	60.3%	03.8%	−	0.656*	0.430*	− 11.12*	1.28	0.89^‡^	0.10

Descriptive statistics, CFA, and IDP were computed on the overall sample (*N* = 446), ITS was calculated on a random sample of 40 participants

M = mean; Medn. = median; SD = standard deviation; SK. = skewness; K. = kurtosis; %Min = percentage of subjects who choice the first category of response (1 = strongly disagree); %Max = percentage of subjects who choice the last category of response (5 = strongly agree); F1 = Self-D; F2 = FES; *λ* = factor loadings; *R*^2^ = explained variance; *t*_*i*_ = independent sample *t*-test; *t*_*p*_ = paired sample *t*-test; *d* = effect size (Cohen’s d)

*p-value < 0.001; ‡ *p*-value > 0.05, *ns*

Results from the CFA suggested a non-adequate two-related-factors solution for the Italian WSSQ: the *χ*^2^ was statistically significant [SB*χ*^2^ (53) = 231.219; *p* < 0.001] and both the RMSEA and the CFI did not meet the recommended threshold suggesting a non-ideal fit: RMSEA = 0.087; CFI = 0.887, despite an acceptable SRMR (0.066).

Exploration of modification indexes [45, 46, 49, 69] also revealed that the model could be improved by correlating residuals of item#10 and item#11 (Δ*χ*^2^ = 41.345). More in detail, a deep examination of these items suggested a possible *non*-independence among these two indicators. After correlating these items’ residuals [45, 46, 49, 69], the model provided a better fit to the data (*r*_item#10-#11_ = 0.396). Despite the Chi-square resulted statistically significant [SBχ^2^ (52) = 189.874; *p* < 0.001], the remaining fit indices overcame the threshold for good model fit: RMSEA = 0.077 [90% CI: 0.065–0.089], CFI = 0.913 and SRMR = 0.066.

As reported in Table 3, each item loaded significantly on its associated factor: mean_loadings_ = 0.64; SD_loadings_ = 0.15; ranging from 0.338 (item#1) to 0.856 (item#4). In addition, a moderate correlation between the two factors was found: *r* = 0.513, *p* < 0.001. Lastly, item’s explained variance (*R*^2^) ranged between 0.114 (item#1) and 0.733 (item#4) with a mean_loadings_ of 0.43 and a SD_loadings_ equal to 0.18.

### Measurement invariance

*Configural invariance*. A configural invariance model was specified: the same two-factor model was estimated simultaneously within each group. A good model fit was found for: (A) gender, (B) BMI status, (C) age, and (D) previous hospitalization for obesity (Table 4). Achieved configural invariance suggests that the factor structure was similar across groups.

**Table 4:** Study2: Goodness-of-fit indices of the WSSQ model and measurement invariance across gender, BMI-status, age and previous hospitalization for obesity

	SBχ^2^ (*df*)	Model H_0_ *LL* (#*fp*)	H_0_ *LL* scaled factor	Scaled -2Δ*LL* (Δ*fp*)	Exact *p*-value	RMSEA	ΔRMSEA	CFI	ΔCFI	SRMR	ΔSRMR
Gender											
Male	93.579 (52)*	− 3030.248 (38)	1.0744			0.069		0.931		0.071	
Female	143.365 (52)*	− 5038.116 (38)	1.0566			0.080		0.908		0.069	
Configural Inv.	237.339 (104)*	− 8.068.364 (76)	1.0655			0.076		0.917		0.070	
Metric Inv.	250.877 (114)*	− 8.074.929 (66)	1.0691	12.604 (10)	0.247	0.073	0.003	0.915	0.002	0.075	− 0.005
Scalar Inv.	287.318 (124)*	− 8.094.538 (56)	1.0751	37.873 (10)	< 0.001	0.077	− 0.004	0.898	0.017	0.077	-0.002
BMI											
Moderate obesity	108.850 (52)*	− 3311.834 (38)	1.0492			0.077		0.908		0.075	
Severe obesity	137.246 (52)*	− 4762.727 (38)	1.0673			0.079		0.914		0.064	
Configural Inv.	245.749 (104)*	− 8.074.561 (76)	1.0582			0.078		0.912		0.069	
Metric Inv.	260.397 (114)*	− 8.081.474 (66)	1.0637	13.530 (10)	0.196	0.076	0.002	0.909	0.003	0.075	− 0.006
Scalar Inv.	278.395 (124)*	− 8.089.979 (56)	1.0777	17.264 (10)	0.069	0.075	0.001	0.904	0.005	0.077	− 0.002
Age											
Younger	94.574 (52)*	− 3298.658 (38)	1.0233			0.068		0.935		0.068	
Older	142.366 (52)*	− 4766.945 (38)	1.0922			0.081		0.906		0.071	
Configural Inv.	237.057 (104)*	− 8065.603 (76)	1.0578			0.076		0.918		0.070	
Metric Inv.	258.628 (114)*	− 8076.489 (66)	1.0640	21.411 (10)	0.018	0.075	0.001	0.910	0.008	0.078	− 0.008
Scalar Inv.	–	–	–	–	–	–	–	–	–	–	–
Previous hospitalization											
No	148.807 (52)*	− 4471.445 (38)	1.0643			0.087		0.892		0.076	
Yes	105.144 (52)*	− 3615.957 (38)	1.0645			0.071		0.924		0.068	
Configural Inv.	253.598 (104)*	− 8087.402 (76)	1.0644			0.080		0.906		0.072	
Metric Inv.	262.544 (114)*	− 8091.430 (66)	1.0648	7.587 (10)	0.669	0.076	0.004	0.907	− 0.001	0.075	− 0.003
Scalar Inv.	273.640 (124)*	− 8096.161 (56)	1.0758	9.432 (10)	0.492	0.074	0.002	0.906	0.001	0.077	− 0.002

χ^2^ = Chi-square test; *df* = degree of freedoms; *LL* = Log-Likelihood test; *fp* = free parameters; Δ(…) = differences between indices; RMSEA = Root mean square error of approximation; CFI = Comparative fit index; SRMR = Standardized root-mean square residual

*p < 0.001

*Metric invariance*. Equality of item factor loadings across groups was examined in a metric invariance model: all factor loadings were constrained to be equal across conditions. The metric invariance model well fit the data (Table 4). Compared to the configural model, no significant decrease in fit indices was found for (A) gender [− 2Δ*LL* (10) = 12.604; *p* = 0.247; ΔRMSEA = 0.003; ΔCFI = 0.002; ΔSRMR = -0.005], (B) BMI [− 2Δ*LL* (10) = 13.530; *p* = 0.196; ΔRMSEA = 0.002; ΔCFI = 0.008; ΔSRMR = -0.006] and (D) previous hospitalization for obesity [− 2Δ*LL* (10) = 7.587; *p* = 0.669; ΔRMSEA = 0.004; ΔCFI = -0.001; ΔSRMR = -0.003]. Inversely, a significant loss of fit compared to the configural model was found for (C) age in the Log-likelihood rescaled difference test [− 2Δ*LL* (10) = 21.411; *p* = 0.018]. Achieved metric invariance indicated that the items were equivalently related to the latent factor in each group.

*Scalar invariance*. Equality of item intercepts was examined in a strict invariance model: all factor loadings and all item intercepts were constrained to be equal across groups. The model well fitted the data for each condition except for gender (CFI = 0.898). No decrease in fit indices compared to the metric invariance model was found for (B) BMI [− 2Δ*LL* (10) = 17.264; *p* = 0.069; ΔRMSEA = 0.001; ΔCFI = 0.005; ΔSRMR = − 0.002] and (D) previous hospitalization for obesity [− 2Δ*LL* (10) = 9.432; *p* = 0.492; ΔRMSEA = 0.002; ΔCFI = 0.001; ΔSRMR = − 0.002]. Conversely, a significant model decrease in fit was observed for (A) gender [− 2Δ*LL* (10) = 37.873; *p* < 0.001; ΔRMSEA = − 0.004; ΔCFI = 0.017; ΔSRMR = − 0.002]. As scalar invariance was achieved, it indicated that groups had the same expected item response at the same absolute level of the trait.

### Psychometric properties

The analysis of IDP (reported in Table 3) revealed that each of the 12 items well discriminates between subjects without IWS from individuals with IWS—with a high magnitude (*d*) in all cases. More in detail, the absolute values of the discrimination parameter (*t*-test: *t*_i_) ranged from |8.57| (item#2) to |25.31| (item#7), with an associated effect size that ranged respectively from 1.00 (item#2) to 2.90 (item#7)—mean_*t*_ = -15.71; SD_*t*_ = 5.21; mean_*d*_ = 1.82; SD_*d*_ = 0.60. With no exception, all the discriminating values suggest a strong relationship between each of the WSSQ item and the underlying IWS construct.

Temporal stability and test–retest reliability provided good results: Self-D, ICC_(T1,T2)_ = 0.883 (95% CI: from 0.779 to 0.938), *p* < 0.001; FES: ICC_(T1,T2)_ = 0.852 (95% CI: from 0.721 to 0.922), *p* < 0.001; Total score: ICC_(T1,T2)_ = 0.886 (95% CI: from 0.785 to 0.940), *p* < 0.001).

Moderate-to-large bivariate correlations indicated that both WSSQ factors and the WSSQ total score were positively and significantly associated with WBIS, CES-D, and BUT. A significant small correlation was also found between BMI and FES factor, whereas—in line with previous literature—no significant correlation was found between WSSQ and BMI for the Self-D subscale and the total score (Table 5).

**Table 5:** Study 2: correlations between measures

		M	SD	1	2	3	4	5	6	7	8	9	10	11	12
1	WSSQ—Total	31.62	9.89	–											
2	WSSQ—Self-D	18.02	5.89	0.865*	–										
3	WSSQ—FES	13.59	5.78	0.875*	0.513*	–									
4	WBIS	42.54	14.48	0.665*	0.548*	0.615*	–								
5	CES-D	31.30	10.84	0.552*	0.472*	0.431*	0.553*	–							
6	BUT—GSI	1.77	1.08	0.722*	0.591*	0.656*	0.740*	0.618*	–						
7	BUT—WP	2.17	1.19	0.650*	0.547*	0.575*	0.674*	0.556*	0.927*	–					
8	BUT—BICons	2.40	1.28	0.725*	0.586*	0.666*	0.752*	0.543*	0.947*	0.876*	–				
9	BUT—A	1.38	1.30	0.708*	0.582*	0.641*	0.649*	0.599*	0.910*	0.773*	0.817*	–			
10	BUT—CSM	1.08	0.87	0.389*	0.279*	0.374*	0.513*	0.451*	0.712*	0.605*	0.585*	0.557*	–		
11	BUT—Dep	1.29	1.36	0.654*	0.541*	0.588*	0.640*	0.606*	0.914*	0.776*	0.817*	0.880*	0.603*	–	
12	BMI	42.28	7.59	0.081†	0.005†	0.134‡	0.025†	0.091†	0.058†	-0.32†	0.082†	0.116†	− 0.052†	0.127†	–

*p < 0.001; ‡ *p* = 0.005; † *p* > 0.05 ns

## Discussion

The alarming rates of obesity have brought widespread attention to the medical consequences of this public health problem [12, 15, 16, 32, 78, 79]. Stigmatization and discrimination are still experienced in different conditions [80–82], and in individuals with overweight/obesity these biases might lead to serious health consequences [17, 18, 26–29]. Interventions aimed to specifically address the IWS are urgently required, and more research is needed to support and improve them [23].

Across studies, languages and cultures [17, 19, 35, 36], the WSSQ was shown to be a valid and reliable tool measuring weight self-stigma [17].

The present study is the first aimed to explore and assess the psychometrical proprieties of the Italian version of the WSSQ in a sample of adult inpatients with overweight/obese. In addition, it is unique in examining the WSSQ measurement invariance across several conditions.

In line with previous researches [83, 84], the first study was aimed to explore the structure of the WSSQ. In line with its original validation study [17], the exploration of the factorial structure of the WSSQ (Study1) indicated the presence of two factors (“Self-devaluation” and “Fear of enhanced stigma”) accounting for 55.04% of variance Moreover, items’ significant cross-loadings were lower than the threshold of 0.3—confirming this factorial solution.

In Study2, the two-factor structure of the WSSQ was further confirmed: results of the CFA revealed that—after correlating residuals for item#10 and item#11—the questionnaire shows a good fit to the data. Moreover, the two-factor solution of the WSSQ—Italian version was in line with previous studies validating the tool in other languages (English [17], German [19], Arabic [35], and French [36]). Each subscale of the Italian WSSQ showed reliability indices over the cutoff value for adequate internal consistency [39]—both in Study1 and Study2—in particular, the Self-D’s alpha was higher than those reported for the German and the Arabic versions of the WSSQ (0.74 and 0.75, respectively), but lower than in its original validation study (0.87). The FES’s alpha showed an almost perfect overlap with the German version (0.83) and it was higher than those of the original and the Arabic versions of the tool (0.81 and 0.78, respectively). The Italian WSSQ also demonstrates good test–retest reliability, with slightly different values from the work of Lillis and colleagues [17].

Moreover, in line with the existing literature, positive and statistically significant relationships were found between both factors of the WSSQ and the convergent measure of weight-related stigma (WBIS), the BUT-body image concerns subscales, and depressive symptomatology (CES-D). Although a non-significant association between Self-D and BMI was detected in the present as in previous research (i.e., German, Arabic, and French versions), it was not in its the WSSQ original validation study [17]. In addition, the correlation between FES and BMI was consistent with the study of Lillis and colleagues [17], but in contrast with other validation studies [19, 35, 36]. These results suggest a potential cross-cultural influence in the relationship between BMI and self-stigma interpretation.

To our knowledge, this is the first study examining the WSSQ measurement invariance across gender, BMI, age, and the occurrence of previous hospitalization(s) for obesity. Age was shown to affect item responses; indeed, only configural invariance was achieved. These results suggest that the factor structure of the WSSQ is the same for both age group, but also that they interpret the items differently and at a different absolute level of the trait. In line with the results of Maïano and colleagues [36], measurement invariance suggested a possible effect of gender on the WSSQ responses: only metric invariance was held, indicating that the same latent factors were measured in each group, but there was a non-equivalence of the trait’s level measured (items intercept).The WSSQ also demonstrated scalar invariance across BMI and the occurrence of previous hospitalization(s) for obesity, indicating the equivalence of the factor structure model, item factor loadings, and item intercepts across these groups. These results showed that—within these two variables—the observed differences in item means (between groups) are due to factor mean differences only.

Among, the limitations of the study we consider the self-report nature of selected measures—whose answers might have been influenced by social desirability and items’ comprehensibility. Moreover, a convenience sample of adult inpatients with overweight and obesity was recruited for this study—and future research should be conducted on outpatients or children/adolescents to confirm the reliability of this findings. Despite a WSSQ post-treatment measure, the small sample size (*n* = 40) prevented from assessing longitudinal MI—the collection of more data at the end of hospitalization could solve this issue.

Despite these limits, the present study is the first consistently increasing knowledge in the field since—compared to previous WSSQ validation studies—the invariance of the questionnaire was tested across several conditions, and the Italian WSSQ can, therefore, be considered a valid and reliable instrument for assessing weight self-stigma in adults with overweight and obesity.

Regarding the implications for the clinical and research field, the WSSQ represents a useful tool that—given its brief and agile administration—it is suitable both for hospital-based [85–87] and at-distance technology-based interventions [88–90] aimed at monitoring and promoting the individuals psychological health over time [91–93].

## Conclusion

The Italian WSSQ is a solid tool for the measurement of IWS and can extend the research concerning stigma in obese people. This is of great importance for improving treatment outcomes in clinical practice, as individuals with high IWS are at greater risk for psychosocial impairments [17, 19, 35, 36, 94] and failure to weight loss/maintenance [23, 95]. The WSSQ can be used to properly identifying those subjects who would benefit from specialized interventions aimed at reducing IWG, also allowing the evaluation of the impact of such treatments.

## What is already known on this subject?

Weight stigma is a widespread complex problem among individuals with overweight and obesity.

The Weight Self-Stigma Questionnaire (WSSQ), is a solid, valid and reliable tool for the measurement of weight-related bias in overweight and obese people.

The WSSQ was successfully translated and/or validated in several languages such as English, German Arabic, and French.

## What does this study add?

The present study aimed to validate the Italian version of the WSSQ in a sample of inpatients with overweight and obesity.

This study for the first time aimed to test—psychometric properties of the WSSQ such as measurement invariance across gender, body mass index (BMI), age, and occurrence of previous hospitalization(s) for obesity.

The factorial structure of the WSSQ was successfully replicated across two independent studies (Study1 and Study2).

## Data Availability

The datasets generated during and/or analyzed during the current study are available from the corresponding author on reasonable request.
